# A review of new hormonal therapies for prostate cancer in black men: is there enough data?

**DOI:** 10.1186/s12885-020-07780-7

**Published:** 2021-01-14

**Authors:** Matthias E. Meunier, Pascal Blanchet, Yann Neuzillet, Thierry Lebret, Laurent Brureau

**Affiliations:** 1 Department of Urology, Foch Hospital, 40 rue Worth, 92150 Suresnes, France; 2 University of Versailles-Saint-Quentin-en-Yvelines, Versailles, France; 3 Department of Urology, Pointe-à-Pitre University Hospital, Pointe-à-Pitre, Guadeloupe France; 4 Inserm, UMR_S 1085 - IRSET, Pointe-à-Pitre, Guadeloupe France

**Keywords:** Abiraterone, Apalutamide, Black men, Enzalutamide, Hormonotherapy, Prostate Cancer

## Abstract

**Background:**

Prostate cancer among black men is known to have specific molecular characteristics, especially the androgen receptor or enzymes related to the androgen metabolism. These targets are keys to the action of new hormonal therapies. Nevertheless, literature has a lack of data regarding black men. We aimed to gather the available literature data on new hormonal therapies among black populations.

**Methods:**

We conducted a literature review from the PubMed / MEDLINE database until October 2020. All clinical studies of new hormonal therapies and black populations, regardless of methodology, were included.

**Results:**

Four studies provided data on new hormonal therapies in black populations. Three studies reported a PSA decline in black patients treated with Abiraterone, higher in black men than in white men. Overall survival also appears to be higher in black patients treated with Abiraterone only or first.

**Conclusion:**

Few articles have evaluated the effectiveness and safety of use of these treatments among black populations. The first results seem to show that Abiraterone can provide a benefit in overall survival in black populations. Prospective studies are needed to answer these questions in the future.

## Background

Prostate cancer (PCa) is the second most common cancer and the fifth most deadly cancer in men worldwide [1]. The effect of castration on metastatic PCa by androgen injections has been known since the 1940s [2]. It has been since then the cornerstone of metastatic PCa care.

In the early 2000s, the only treatment option for metastatic castration-resistant prostate cancer patients (mCRPC) was chemotherapy. Since then, new hormonal therapies (i.e Abiraterone, Enzalutamide and Apalutamide) have become the new standard of care for patients with advanced or metastatic PCa. They have demonstrated their efficacy and safety, first in metastatic castration-resistant prostate cancer patients (mCRPC) [3, 4], then in patients with metastatic hormone-sensitive prostate cancer (mHSPC) [5–7] and finally in non-metastatic patients castration-resistant patients (M0 CRPC) [8, 9]. In addition to their simplicity of use related to their oral form and an acceptable tolerance profile, these treatments have shown a significant survival gain at advanced and metastatic stage.

Prostate cancer among black men is known to have specific molecular characteristics [10], especially the androgen receptor (AR) or enzymes related to the androgen metabolism (i.e CYP17). These targets are keys to the action of new hormonal therapies. Nevertheless, literature has a lack of data regarding black men. A recent analysis of 1,319,225 patients from the National Cancer Database (NCDB) demonstrates no racial survival disparities for African American and white men with bone, liver, lung, or brain metastasis [11]. Nevertheless, due to the lack of treatments related data, no conclusion could be drawn regarding the efficacy of new hormonal therapies in the black population.

We aimed to gather the available literature data on new hormonal therapies among black populations.

## Methods

### Research strategy

We conducted a literature review from the PubMed / MEDLINE database, with no date restrictions until october 2020, using the following keywords: “Abiraterone”, “Apalutamide”, “Darolutamide”, “Enzalutamide” and “African men”, “Black men”. Due to the small number of studies published in English, all clinical studies of new hormonal therapies and black populations, regardless of methodology, were included. Abstract and reviews of literature have been excluded. The main outcomes were: the decline of prostate specific antigen (PSA), defined as efficacy or antitumor activity in the reported studies, survival results (overall survival and progression-free survival) and reported adverse events.

## Results

Four studies were found, published between December 2016 and November 2019, evaluating the PSA decline, overall survival, progression-free survival and adverse events of new hormonal therapies. The characteristics of the patients included in these studies are summarized in Table 1. In total, *n* = 1191 patients were included in these studies, including 1116 patients for the retrospective cohort by Leuva et al. Three studies evaluated abiraterone only, and one abiraterone and/or enzalutamide with mostly abiraterone treated patients. Three of the four studies evaluated patients with mCRPC, and one, patients with M0 CRPC. The median age of patients, when available was between 66 and 78 years. The grade groups, when available, where split in half between patients with low risk to intermediate risk (i.e. grade groups for Gleason score 1 to 3) and patients with high risk (i.e. grade groups for Gleason score 4 and 5). Most patients where asymptomatic and had a good performance status. One study was a large retrospective cohort, two were single-arm phase II studies with a prospective design, and one was a retrospective, comparative case-control study, with a level of evidence of respectively 2b, 2c, 2c and 4, according to the levels of evidence for therapeutic studies from the centre for evidence-based medicine [16].

**Table 1:** Characteristics of selected trials

Trial (NCT)	Study design	Level of evidence	Treatment	Population	Number of patients (N)	Median age	Baseline PSA	Gleason grade groups	PS
Tsao et al. 2016 NCT01735396 [12]	Ph. II prospective study	2c	Abiraterone	mCRPC black men	11	66	NR	NR	OMS 0 73% OMS 1 27%
Ryan et al. 2018 NCT01314118 [13]	Ph. II prospective study	2c	Abiraterone	M0 CRPC	19	NR	NR	NR	NR
Ramalingam et al. 2017 [14]	Retrospective comparative case-control study	4	Abiraterone	mCRPC black men	45	NR	NR	Gr. 1–3 42% Gr. 4–5 58%	OMS 0 92% OMS 1 8%
Leuva et al. 2019 [15]	Retrospective cohort study	2b	Abiraterone and Enzalutamide	mCRPC black men	1116	73–78	45.8–48.7	NR	NR

*NR* Not Reported

### PSA decline

The only prospective single center study for patients with mCRPC demonstrated a 90% antitumor activity (defined as a 30% PSA level decline) of the Abiraterone among black men [12]. A retrospective comparative study showed a higher antitumor activity in black men with mCRPC than white men: statistically significant differences in favor of Black men were found in the proportion of patients achieving a 50% PSA level decline (Black men 68.9%, White men 48.9%, *P* = 0.028) and 30% PSA level decline (Black men 77.8%, White men 54.4%, *P* = 0.008). Rates of primary Abiraterone-refractory disease (PSA increase as best response) also trended to be higher in White men (31.1%) than in Black men (15.6%) patients (*P* = 0.052) [14].

Similarly, Leuva et al. found among a large sample of nearly 1000 black patients versus three thousand white patients that Abiraterone efficacy was 60% higher in black men than in white men, using a novel approach based on the growth and regression theory, using serial PSA values (*p* = 0.02). On the other hand, no difference between black and white patients treated with Enzalutamide was demonstrated [15].

### Survival outcomes

Leuva et al. demonstrated that the overall survival of black patients was statistically significantly higher than that of white patients, with 3 months of survival benefit (25.4 months versus 22.4 months, *p =* 0.02) in patients treated with Abiraterone only or with Abiraterone first (followed by Enzalutamide). These differences were unaffected by prior Taxane chemotherapy exposure. No difference of survival was reported between black and white patients treated with Enzalutamide [15].

A prospective comparative study between black and white men with M0 CRPC did not find any differences for PSA progression-free survival (median: 29.0 vs. 28.6 months, *p* = 0.57) and radiological progression-free survival (median: 41.4 vs not reached, *p* = 0.82) [13].

A retrospective comparative study also did not find any survival differences between black and white men with mCRPC: Median overall survival (Black men 27.3 months, White men 24.8 months, *P* = 0.669) and median time to PSA progression (Black men 11.0 months, White men 9.4 months, *P* = 0.917) [14].

### Adverse events

Only one prospective study has fully reported adverse events. No patients discontinued treatment because of adverse events and treatment was well tolerated. Yet, Higher common events were reported than expected. For example, the authors report fatigue in 70% of patients, which is higher than the phase III studies carried out in a predominantly Caucasian population [4, 17]. All these results are presented in Table 2.

**Table 2:** Outcomes of selected trials

Trial (NCT)	Population	Comparison	Primary Outcomes	Secondary Outcomes	Results	Adverse Events
Tsao et al. 2016 NCT01735396 [12]	mCRPC black men	–	Antitumor activity (defined by a 30% decline in PSA level)	–	90% antitumor activity	No patients discontinuing treatment because of AE Higher common AE
Ryan et al. 2018 NCT01314118 [13]	M0 CRPC black men	mCRPC white men	Rate of 50% PSA decline	Time to PSA and to Rx progression	Primary: No data Secondary: No difference	NR
Ramalingam et al. 2017 [14]	mCRPC black men	mCRPC white men (2 W:1B)	Rate of 90, 50 and 30% PSA decline	Time on therapy, time to PSA progression, OS	Primary: 90% PSA did not differ between B and W, but 50 and 30% decline were significantly higher among B Secondary: time on therapy, time to PSA progression and OS did not differ between B and W	NR
Leuva et al. 2019 [15]	mCRPC black men	mCRPC white men	Overall survival	Efficacy of treatment using tumor growth and regression, were calculated using serial PSA values	B Patients treated with Abi only or with Abi first showed superior survival than W patients: 25.4 months vs. 22.4 months (*p =* 0.02) Abi efficacy was 60% higher in B than in W patients (*p* = 0.02) No difference between B and W patients treated with Enza	NR

*B* Black, *W* White, *NR* Not Reported, *AE* Adverse events, *Abi* abiraterone

* Theory for growth and regression, that uses a novel set of equations, was validated in *>* 20,000 patients. *G w*as an excellent biomarker of overall survival

## Discussion

New hormonal therapies have been a revolution in the management of advanced and metastatic prostate cancer. Numerous large-scale prospective studies have shown both the efficacy and safety of these treatments. However, most of these multicentre studies have been conducted in North America, Europe or Asia, where populations contain only few black subjects [3–9]. In addition, ethnicity data of these patients is not specified. In practice, the new hormonal therapies are the reference treatment for prostate cancer, including for black populations, despite molecular specificities interfering with the mechanisms of action of the molecules used (i.e Abiraterone, Enzalutamide, Apatumide and Darolutamide).

Abiraterone is an androgen biosynthesis inhibitor, that inhibits 17 a-hydroxylase/C17,20-lyase (CYP17). The results of meta-analysis suggest that *CYP17* polymorphisms may have a role in PCa susceptibility in African Americans [18, 19]. Notably, rs743572 polymorphism seems to be associated with a higher PCa risk in the black population, but not in Caucasian or Asian population [20]. Mutations in genes encoding the target might lead to a different efficiency of the molecule in the black population.

Enzalutamide as well as Apalutamide and Darolutamide are AR inhibitors that inhibit multiple steps in the AR signaling pathway. Comparing mutations of 200 black men versus 100 white men, Koochekpour et al. found that somatic missense AR mutations were detected at a higher rate in black men (17 out of 200 cases) than in white men (2 out of 100 cases). Analysis of genomic DNAs extracted from white blood cells of patients with sporadic PCa revealed that the rate of germline AR mutations were also 4 times higher in black men than in white men [21]. Gaston et al. found that AR protein expression was 81% higher in localized PCa of black men compared to white men [22]. Even though, due to a lack of data, no difference was found in our review for these new AR inhibitors, AR mutations and expression could result in a differentiated effect of these treatments depending on race.

Only four studies were found to provide data on new hormonal therapies in black populations, including three study for Abiraterone only, and one large scale study with 90% treated with Abiraterone only or Abiraterone first. These studies have showed a real PSA decline with Abiraterone, which seems to be higher in black population than in white population. Concordantly, Leuva et al. found that Black men treated with Abiraterone only or first, presented a higher overall survival. Molecular specificities of prostate cancer among black men might allow a better efficacy of Abiraterone in this population.

*NCT01940276* is a prospective multicenter trial currently underway, comparing black and white men with mCRPC receiving Abiraterone. The first results are similar to previous findings, showing that PSA progression-free survival was higher among black men than white men [23]. McNamara et al. also recently reported retrospective data comparing mCRPC chemotherapy-naïve black (*n* = 787) and white patients (*n* = 2123). Black men had better overall survival than white men with 918 days and 781 days respectively (HR = 0.826; 95%CI [0.732–0.933]) [24]. These preliminary data seem to confirm the survival benefit for black patients with mCRPC treated with Abiraterone.

Several prospective studies are currently evaluating Abiraterone, Enzalutamide or Apalutamide as well as combinations, focusing on black populations. None were found in this population on Darolutamide. We present a summary of ongoing prospective studies in Table 3.

**Table 3:** Ongoing prospective studies, evaluating new hormonal therapies in black populations

Trial (NCT)	Study design	Treatment	Population	Number of patients (N)
NCT02415621	Phase I	Abiraterone	mCRPC	10
NCT03770455	Phase II	Abiraterone or Enzalutamide + Avelumab	mCRPC	27
NCT01940276	Phase II	Abiraterone	mCRPC	50
NCT03098836	Phase II	Abiraterone + Apalutamide	mCRPC	50

Regarding adverse events, few data are available. Even though in the Tsao et al. study incidence of some common adverse effects may have been higher than expected due to small sample size, adverse events seemed to be similar by frequency and severity by race in the first results of the prospective trial *NCT01940276* conducted among black and white subjects [23].

However, these studies are insufficient and large multicenter prospective studies among black populations, with Caucasian control group, would be needed to specifically address the questions of efficacy, survival benefit and security of treatment with new hormonal therapies in black men, especially regarding Abiraterone.

## Conclusion

After an inventory of the literature, few articles have evaluated the effectiveness and safety of use of these treatments among black populations. The first results seem to show that Abiraterone can provide a benefit in overall survival in black populations. New large prospective studies are needed to answer these questions in the future.

## Data Availability

Not applicable.

## Abbreviations

AR: Androgen receptor
M0 CRPC: Non-metastatic patients castration-resistant patients
mCRPC: Metastatic castration-resistant prostate cancer patients
mHSPC: Metastatic hormone-sensitive prostate cancer
PCa: Prostate cancer
PSA: Prostate-Specific Antigen
